# "I should have discharged him but I felt guilty": a qualitative investigation of clinicians' emotions in the context of implementing occupational therapy

**DOI:** 10.1186/s13012-014-0141-9

**Published:** 2014-10-02

**Authors:** Niina Kolehmainen, Jennifer McAnuff

**Affiliations:** Institute of Health and Society, Newcastle University, Newcastle, UK; Community Children's Occupational Therapy, Leeds Community Healthcare NHS Trust, Leeds, UK

## Abstract

**Background:**

Clinicians' emotions about practice are a potentially powerful yet largely overlooked factor in implementation of good-quality care. The present paper expands the current, limited evidence about clinicians' emotions by (i) describing clinician-reported examples of emotions about practice and (ii) identifying the clinical situations in which, according to clinicians, emotions emerge and influence practice.

**Methods:**

Semi-structured, face-to-face interviews with 25 clinicians (children's occupational therapists) were conducted across six health care organisations. Participants were asked to reflect on their practice in two recent patient cases, one that they perceived ‘successful’ and another ‘unsuccessful’. Interviews were transcribed *verbatim*, and the transcripts were analysed for emerging themes. A proportion of transcripts were independently read and coded, and the themes were validated through critical discussion.

**Results:**

A key theme was clinicians’ emotions, especially negative emotions including guilt, anger, worry, frustration and inadequacy. These were described in connection with situations where the clinicians perceived that (i) they failed to provide good quality care, (ii) they were unable to achieve positive health outcomes or engage the patient or (iii) there was conflict between what they were asked to do and the norms they held important.

**Conclusions:**

Clinicians experience a range of negative emotions about practice. These are particularly likely to emerge in situations where clinicians perceive that their actions and practice fall short of the standards, norms or outcomes that they hold as important. The results inform the specification of emotions and emotion-triggering situations for future investigations of health care implementation.

## Background

Rehabilitation clinicians' delivery of good-quality care can be influenced by a wide range of factors. A recent review of frameworks about factors influencing delivery of care [1] lists 57 clusters of factors, some of which (e.g. clinicians' knowledge and skills [2]) have already received considerable research attention. Strategies to facilitate delivery of good-quality care have also been identified (e.g. educational materials [3], and audit and feedback [4]). However, a substantial proportion of the variation in clinicians' delivery of care remains unexplained [5], and the effects of known strategies for improving the quality of care are small [3],[4]. There is a continuing need to identify more effective ways to improve health care delivery, including delivery of rehabilitation care. One part of addressing this need is to investigate under-researched factors related to clinicians' practice and delivery of good-quality care. The results of such studies can be used to identify new strategies that are as yet untested [6].

One under-researched factor is clinicians' emotions concerning their practice and delivery of care [5]. For example, a search of Implementation Science in July 2014 for papers with titles including any of the terms ‘emotion’, ‘feel’, ‘feeling’ or ‘affect’ yielded no papers, and the UK National Institute for Health and Care Excellence guidance [7] on changing professionals' practice makes no reference to clinicians' emotions. Considering that positive and negative emotions are known determinants of people's actions in general [8]-[10], and that clinicians are people, it is plausible that clinicians' emotions may play a role in their practice. This hypothesis is also supported by the few studies that have included clinicians' emotions among the factors that they have investigated. A study of primary care practitioners [11] identified clinicians' feelings of embarrassment as a possible determinant of the clinicians' avoidance of discussions about sexual health with patients, and a study of mental health workers [12] identified that clinicians' feelings of fear and wariness were a possible determinant of their reluctance to provide treatment to patients. A small number of studies with medical doctors have also found that clinicians' negative emotions concerning a perceived medical error can affect their future practice, sometimes for years after the event that initially aroused the emotions [13]-[16].

While the evidence described above provides support for the general hypothesis about the role of clinicians' emotions, it provides little evidence about the nature and context of the emotions. In other words, questions about *what emotions* clinicians feel in implementing care (e.g. happiness, satisfaction, anger) and *what situations* trigger these emotions (e.g. patient encounters, team interactions) remain largely unanswered. Yet, it is this specific evidence that is required for emotions to be considered as a target for implementation interventions. Early evidence of this kind is typically obtained from open-ended, qualitative studies. However, qualitative studies of clinicians' emotions have so far largely focused on emotions as `emotional labour' or `emotional skills' [17],[18], that is, as something used to manage other people's (usually patients') emotions and behaviours. The aims of the present paper are to (i) provide examples of the *nature* of clinicians' emotions and (ii) identify clinical *situations* in which, according to clinicians, emotions emerge. This evidence about the phenomenon (i.e. what emotions clinicians feel and in what situations these emotions emerge) contributes to developing theory about clinicians' emotions in the context of implementation of good care. Developing such a theory is the recommended first step to developing effective implementation interventions [6],[19].

## Methods

This was a qualitative, semi-structured, face-to-face interview study that was part of a wider mixed methods programme of research [20] about clinicians' practice in one clinical speciality (paediatric occupational therapy). The programme of work (i) identified factors related to clinicians' practice that may explain—qualitatively or quantitatively—variation in the quality of patient care and (ii) designed an intervention to target these factors. The present qualitative study was part of the identification of factors and focused on generating evidence about clinicians' views, their practice, and the context of their practice including any emerging, previously unidentified issues. The programme of work adopted a pragmatic [21] perspective on evidence; this meant identifying `what might be important and might work' [21],[22] in the context of applied clinical practice. As the first exploratory step of the programme, the present study used an inductive approach—that is, we used the data to identify themes and categories, and then compared these with existing literature to identify relevant theories and frameworks that could be used in subsequent steps (see also ‘Discussion’). The programme of research had National Health Service (NHS) Research Ethics Committee approval (07/S0801/55).

### Sampling and recruitment

The participants in the programme of research formed the sampling frame for the present study. The programme of research involved a random sample of six mainland NHSScotland occupational therapy children's services, and 26 randomly sampled clinicians (registered occupational therapists) within them. This covered 55.5% (6/11) of the NHS organisations and 19.0% (26/137) of NHS children's occupational therapy posts in Scotland. Research and routine data in children's therapy services in the United Kingdom, including Scotland, is overall very limited [23], and there were no bases for theoretical or purposive sampling.

All six services and the 26 clinicians participating in the programme of research were invited to participate in the present interview study by sending them a letter and an information leaflet about the study. Furthermore, within clinicians, purposive sampling was used to collect a range of examples of practice situations from each participant. This involved asking each participant to select two contrasting, recently discharged cases (one that the participant considered ‘successful’ and another that they considered ‘unsuccessful’) from their caseloads.

There is no single, established approach to deciding a sample size in qualitative studies [24]. This reflects the nature of qualitative research: the focus is on open-ended discovery where the researcher follows emergent empirical and conceptual findings and may not know, in advance, how much and what data they will need [24]. For the present study, we emphasised (i) inclusion of participants across services in order to investigate the topic across a range of clinical, organisational, geographical, social and team contexts and (ii) inclusion of a sufficient yet feasible number of clinicians (*n*?=?15-26) in order to enable identification of differences and similarities in views between clinicians. This approach reflects current thinking about sample size considerations in qualitative research [24].

### Procedures and materials

A topic guide based on the critical incident technique [25] was used to elicit participants' views. Critical incident technique is a well-established interview method for eliciting people's views and experiences in relation to a phenomenon (here, delivery and clinical management of patient care) and ‘incidents’ within it (here, clinicians' actions/practices and related thoughts and feelings). The interviews were initiated by asking participants an open-ended question: "[could you] describe and reflect upon [your chosen] case and the care process, the actions and decisions you took, and the events that followed" [11]. Participants commonly responded by providing (i) a summary description of the characteristics of the child and the environment and (ii) a comprehensive narrative about the care process. Further open-ended prompts were used to encourage participants to elaborate and expand on their responses [26]. The interviews were conducted at participants' places of work by the first author, recorded *verbatim* and later transcribed.

### Data analysis

The data were analysed for emerging themes [27],[28]. The first author (NK) identified themes and sub-themes that described the data or underlying ideas in it and assigned the themes descriptive labels. Visual representations [27],[28] were used to explore and identify associations between themes and to develop conceptual categories. Each transcript was first analysed individually, and then themes were identified across transcripts. A second researcher (LM, see ‘Acknowledgements’) independently coded four transcripts and the researchers discussed and further developed the themes. For the present paper, a third researcher (JM) accessed the data related to the theme `negative feelings’ and critically appraised the issues within the theme. Text management software (NVivo v7 [29]) was used for data management.

### Quality assurance

A range of recommended quality assurance techniques were employed to ensure credibility, transferability, dependability and confirmability [26].

#### Credibility

? Prolonged engagement: the lead researcher (NK) had a prolonged engagement with the participating services and clinicians through the wider programme of research; this included informal interactions as well as the research contact. In addition, both authors of the present paper are occupational therapy clinicians and have an understanding of the wider NHS and children's services cultures. The data disclosed by participants indicated a rapport and trust between the lead researcher and the participants—the participants disclosed personal views as opposed to `toed the party-line’ (see, e.g. `Results’ below and a related publication [30]).

? Triangulation: accounts between participants and across a range of services and contexts were compared and contrasted, and three analysts with different theoretical perspectives were involved (NK: professionals' practice, family-centred care, and behaviour change; JM: clinical practice and NHS management; LM: sociology, and organisational and management research).

? Member checking: the themes and their content were shared and discussed with the participants at specific sessions and with the wider clinical community at national and international conferences, meetings and training events.

? Frequent de-briefing: study progress, methods, emerging themes and any issues were reported to and scrutinised by the research programme senior team at regular intervals.

#### Transferability

? Sampling across contexts: please see above `Sampling and recruitment’

? Reporting of the sampling frame and criteria (see above) and the key population characteristics (see `Results’). The depth of reporting on participant and service characteristics had to be balanced with the Research Ethics requirements for protecting participant anonymity.

#### Dependability and confirmability

? Involvement of researchers independent of the initial study: involvement of the third researcher (JM) in the data analysis and peer examination throughout the critical discussion, and regular reporting to the wider programme senior team not involved in the qualitative stream of inquiry.

? Audit trail: the lead researcher (NK) kept field notes and a logbook of data analysis and established an electronic data analysis and synthesis trail of the development of the themes (in NVivo, see above).

? Triangulation: see ‘Credibility’ above.

? Reflexivity: the lead researcher (NK) and the multiple-investigator team reflected on the role of the researcher and these were recorded in the researcher's notes and logbook (see above).

## Results

All 26 clinicians agreed to participate. Due to scheduling difficulties, only 25 were interviewed. All participants were of senior grade, had been qualified for a substantial length of time (median 12 years, interquartile range [IQR] 9-20) and had several years of experience working with children (median 8 years, IQR 6-13).

Several themes emerged from the transcripts. One of the key themes was "negative emotions" and is reported here—other themes ("beliefs about responsibilities", "aims of therapy" and "structure of the therapy process") have been previously reported elsewhere [30]. Examples of clinicians' positive emotions were actively sought from the transcripts; however, descriptions of positive events were not accompanied by clinicians' expressions of their emotions.

Within the theme of negative feelings, four clusters emerged. The first three related to the types of emotions and circumstances in which these emotions emerged: (1) guilt and anger in relation to perceived failure, (2) worry and ‘niggle’ in relation to possibility of failure and (3) frustration and inadequacy in relation to perceived failure to achieve positive outcomes. The content of these clusters was highly consistent across contexts and clinicians. The fourth cluster, (4) emotions and practice, was more tentative—it was covered in fewer interviews and the narratives between clinicians diverged. The four clusters are elaborated on below.

### Guilt and anger related to perceived failure

Participants described feelings of guilt and anger in relation to their perceptions that either they or the service had failed to provide a level of care that they thought desirable. The desired level could be based on the clinician's implicit personal or moral norms, a perceived professional duty or other perceived expectations. The following quotation illustrates a situation where adhering to (an evidence-based) service policy was related to a clinician feeling guilty about falling short of her personal norms.

*NK: "Would you elaborate on what****you said that you felt guilty****in that meeting?"*

*Participant A, service 4: "… (…) I feel****like I'm just making things up****when I say `Well we don't do that, she doesn't need it at this time' (…) and then you've got the eyes of the school, and the mum all looking at you sort of saying ‘Well why are you not coming in’, and****your answer just sounds quite pathetic****(…)."*

The nature of clinicians' feelings appeared to relate to the reason they perceived to underpin the shortcoming. Guilt was described in relation to clinicians' own actions or thoughts; frustration and anger were described in relation to perceived failures on the part of others. The next quotation captures an example of the latter as one clinician discusses local service arrangements for children with intellectual disabilities.

*Participant B, service 3:****I feel enraged****and I have done for years but what do I do? Set up my own [practice]? [I am] Not a psychiatrist. (…) child and family psychiatry is a really good, comprehensive, cohesive service, [they] just would not consider a child with learning difficulties and these are the children of families that are struggling the most.*

### Worry, ‘niggle’ and fear related to possibility of failure

Participants expressed feelings of worry, ‘niggle’ and fear in relation to the *possibility* of failure. Worry was especially discussed in relation to discrepancies between service policies and clinicians' personal norms. For example, participants who described being faithful to their personal norms, rather than service policies, expressed worry about the discrepancy between their practice and the policies.

*Participant C, service 4: "(…)****I'm worried****that I'm; not doing too much but (…) I kind of feel in a way one's being a bit indulgent.. but that's just ridiculous because it's not that but (…)"*

Feelings of ‘niggle’ and fear of possible failure were expressed in relation to situations where clinicians tried to follow the service's recommended practices which differed from their preferred practices.

*NK: "Could you discuss that****`niggling’ feeling****[you mentioned]..?"*

*Participant D, service 2: "(…) I wanted to try to stick to [the clinic] approach and try to be very (…) focussed on problem solving rather than getting involved in things (…)****I suppose [the ‘niggling’ feeling] was just (…) because I've been used to working [in another] way****."*

*Participant E, service 6: "…I suppose it's****the fear of missing something****isn't it, that you discharge them and something happens and****you haven't really covered it****or****you're not doing something that you should be doing****really because****you're missing some great thing that you should be helping them with****."*

### Frustration and inadequacy related to not achieving positive outcomes

Frustration was expressed in relation to situations where participants had identified potential positive outcomes for the child but, for some reason, were not able to achieve them.

*Participant F, service 6: "(…) it was****hugely, hugely frustrating****, where you can see the potential where you could do things but it's just not (…)"*

Where the failure to achieve positive outcomes was perceived to relate to the child's or parent's refusal to accept interventions, clinicians reported feeling disempowered and inadequate and having self-doubts.

*Participant G, service 4: "(…) [the young person] was very reluctant (…) she would just refuse to do it or she would do it really begrudgingly. (…) it's****made me feel that I've not been doing a very good job****, and I went through quite a long time of (…)****berating myself****and flicking through her notes and thinking (…)****made me feel like a really crap OT****(…)"*

### Emotions and clinical practice

The data indicated that clinicians' emotions were entwined with their own practice and other people's actions. In some examples, the descriptions suggested a straightforward link between emotions and actions—an example is captured in the following.

*Participant B, service 3: "[the child]****really wasn't very easy to like****[…] and part of me****felt a guilt****[…] because****I didn't like [the child] very much and I don't think a lot of other people liked him****, and I think if he was nice family, easy to like, didn't have behavioural problems, would his [care] pathway have been different? I think so. And I don't think these factors should come into it so****I tried to overcompensate****for my personal feeling [by seeing him when I no longer needed to do so]. (…)****I should have discharged****[him] sooner - much, much sooner. But****I felt guilty****."*

From this, it is possible to disentangle the emotions (not liking the child and the family, and feelings of guilt) and the perceived consequence of these emotions (the family receives poorer care than a likable family, and the clinician provides the child more treatment than clinically indicated to compensate for the feelings of guilt). Explicit descriptions such as this were rare however; it was more common for clinicians to imply associations between emotions and practice.

A typical example of the implied association is captured in the quotation below. The clinician reflects on practices at the end of care episodes and implies a link between how the clinician feels about the family, the clinician's desire to avoid feeling inadequate for not achieving positive outcomes for that family and the clinician's general tendency to adopt practices that enable him/her to avoid feelings of inadequacy for not helping families.

*Participant H, service 3: "(…) you become involved with children and****you want to give the best that you can****(…) I****didn't want to just think ‘I haven’t got anything and now I'm walking away'****(…) it's****almost like looking for something****, `there must be something that I can tackle’ that I can say `I’ll help you with that (…)'"*

From the data in the present study, it was not possible to make conclusions about the practices associated with clinicians' emotions. For example, the two examples above suggest that negative emotions—or attempting to avoid negative emotions—might be associated with undesirable practices. However, the data also included examples of negative emotions triggering positive actions and desirable practices. This is illustrated in the final quotation, below, where a clinician describes how her feelings of frustration triggered her to initiate a wider team discussion about the quality of the care provided.

*Participant G, service 4: "…The****consultant****had told [the child about the poor prognosis] but hadn't told the parents. (…) [****the child****became]****really aggressive****(…)****grieving****for the loss (…)****I felt frustrated****because I didn't think that [the team] took on board****the parents(…) needs for support****(…) I really didn't think it was clear enough for the family as to what was going on and what the outcome would be (…) in the end****I had to take it to [the wider team****to address the issue]"*

## Discussion

The present paper reports on (i) the nature of clinicians' negative emotions about practice, including feelings of guilt, worry, inadequacy, frustration and anger, and (ii) three typical practice situations in which, according to clinicians, the negative emotions emerge: the clinician perceives s/he has failed a norm, value or standard they hold as important; the clinician perceives s/he is at risk of failing such a norm, value or standard in the future and the clinician feels unable to achieve positive outcomes for the patient.

The results expand the findings from previous studies and contribute to the field in several ways. One key contribution is to the way clinicians' emotions are theorised and subsequently operationalised for investigations and for implementation interventions. So far, literature in implementation science has identified, at a general level, that emotions may explain practice [11],[12]. In this literature, emotions are conceptualised as evaluations of affective consequences or outcomes of a behaviour, e.g. `discussing sexual behaviours with a patient [the practice action] makes me feel awkward/embarrassed [emotional consequence/outcome]' [11]. This reflects the social cognitive view of affect and behaviour [31],[32]. Social cognitive models are a common approach to investigating clinicians' practice [5]; however, there are significant limitations to their usefulness and a need to explore other approaches [33]. The evidence from the present study indicates that evaluation of consequences and outcomes covers only one aspect of clinicians' lived experiences of emotions. From the present results, a revised conceptualisation of emotions can be proposed: clinicians' emotions emerge from a combination of (i) clinicians' commitment to certain outcomes, standards or norms *and* (ii) a perception that they are failing to achieve these outcomes, standards or norms. Specifying clinicians' emotions in this way mirrors the literature about theories of self-regulation of human emotions and actions [34], including notions that (i) desired standards, norms and outcomes have an important role in the emergence of emotions; (ii) a person's evaluation of the rate of their performance against the desired standards, norms or outcomes produces positive or negative emotions and (iii) emotions `feed forward' to shape future actions [34],[35]. The revised specification has direct consequences for how emotions should be considered for elicitation, observation and measurement in future research. The specification indicates that research into clinicians' emotions should incorporate an investigation of the standards, norms and outcomes they hold as important, where they view themselves in relation to these standards, and how this relates to their feelings about the practices they are expected to do. The specification also indicates a need to expand the theoretical basis informing investigations of clinicians' emotions to include self-regulation as well as social cognitive theories.

Another key contribution of the present study is the identification of specific emotions likely to be relevant to investigations of clinicians' practice and implementation of care. The results from the present study provide an evidence-based description of a range of emotions likely to be relevant to clinicians' practice and a description of the situations where these are most likely to be observable. The results specifically indicate that future studies should pay particular consideration to how clinicians manage their own negative emotions in situations where they are required to consider their practices in the context of (patient or other) outcomes, norms and standards.

The results also highlight challenges for future research investigating clinicians' emotions and implementation practices. First, a key characteristic of the situations in which negative emotions emerged was that the clinician *perceived* a failure. (Whether or not there was also an objectively observed failure is left open.) This is an important finding for future research as it indicates that accurate identification of emotion-inducing situations relies on the clinician's willingness to disclose their perceived failures or perceived anticipated failures to the researcher. Second, the relationships between clinicians' emotions and practices were often implied rather than directly discussed. It is possible that this was because clinicians were hesitant to discuss these issues openly; however, it is equally likely that the relationships between emotions and practice are not easily accessible to clinicians and not easy to articulate'this is a known feature of self-regulatory processes [34]. Together, the first and the second point indicate that while some evidence about the nature, content and context of clinicians' emotions can be elicited by interviews, other modes of data collection may also be required to explore the relationships between emotions and practice. A solution could be, for example, the use of longitudinal, mixed methods data collection across individual clinicians' care delivery situations for a period of time. The results of the present study are of particular importance for planning such investigations as they provide a starting point for specifying the target and context of observations, i.e. the emotions and care delivery situations.

In terms of strengths and limitations, a major strength of the present study was that participants discussed their negative emotions about practice unprompted—this lends support for the credibility and confirmability of the results [26]. Another strength was that the participants were recruited across organisations, supporting relevance of the results across organisations. The main limitations were that the participants came from a single discipline and that the study relied on a single mode of data collection (interviews). The present study does not provide evidence about the direction or nature of relationships between emotions and practice or that clinicians' emotions cause undesirable practice. However, the results contribute to the development of the evidence and theory about the phenomenon of clinicians' emotions in the context of implementation of health care. This is the essential first step for investigating causal processes between clinicians' emotions and practice [6],[19].

## Conclusions

Clinicians experience a range of negative emotions about practice. These are particularly likely to emerge in situations where clinicians' perceive that their actions and practice fall short of the standards, norms or outcomes that they hold as important. The results inform the specification of emotions and emotion-triggering situations for future investigations of health care implementation.

## Authors' contributions

NK designed the study and conducted all data collection, conducted the initial data analysis, and wrote the first draft of the manuscript. NK and JM discussed the data and the first draft of the manuscript, and JM made substantial comments. NK and JM revised the manuscript, both authors providing substantial intellectual contributions on all drafts. Both authors approved the final version of the manuscript and take responsibility for its content.

## Authors' information

NK is a Senior Research Fellow and a practicing Senior Occupational Therapist (Honorary). NK has PhD in Health Services Research; she has led the programme of work within which the described study resides since 2006. JM is Clinical and Professional Lead, and Service Manager, in a children's community occupational therapy. JM has MSc in Professional Health and Care Studies and is an active collaborator on a number of allied health care studies.
